# Diversity and Community Structure Underlie Divergence in Thermal Strategies Across Tropical Elevations

**DOI:** 10.1002/ece3.74025

**Published:** 2026-07-25

**Authors:** Cleber J. N. Chaves, Marília M. Tavares, Gabriel P. Sabino, João Pedro S. P. Bento, Vitor de A. Kamimura, Wagner L. dos Santos, Lucas N. Gonçalves, Karina T. Silva, Juliana L. S. Mayer, Kenneth J. Feeley, Clarisse Palma‐Silva

**Affiliations:** ^1^ Laboratory of Evolutionary Ecology and Plant Genomics, Institute of Biology Universidade Estadual de Campinas (UNICAMP) Campinas São Paulo Brazil; ^2^ Laboratory of Plant Anatomy Institute of Biology, UNICAMP Campinas São Paulo Brazil; ^3^ Postgraduate Program in Plant Biology Institute of Biology, UNICAMP Campinas São Paulo Brazil; ^4^ Department of Biology University of Miami Coral Gables Florida USA; ^5^ Fairchild Tropical Botanic Garden Coral Gables Florida USA

**Keywords:** biotic interactions, climate change resilience, community assembly, elevational gradient, evolutionary ecology, niche partitioning, plant physiology, thermal tolerance

## Abstract

We assessed how thermal tolerance strategies are associated with community diversity and phylogenetic context along a tropical elevational gradient, integrating community‐ and population‐level perspectives by examining a widespread focal species together with its co‐occurring species, and evaluated whether biotic and evolutionary context accounts for variation in thermal niches beyond elevation alone. Between 2023 and 2024, we surveyed vascular plant assemblages across seven sites in the Brazilian Atlantic Forest, spanning sea level to ~2200 m elevation, with emphasis on the bromeliad *Pitcairnia flammea* and co‐occurring monocots, dicots, and ferns. Community diversity, species cover, and phylogenetic structure were quantified for each assemblage, and photosynthetic heat and cold tolerance (T50) and leaf functional traits were measured for *P. flammea* populations and dominant sympatric species. This two‐level design allowed community‐wide patterns among species to be assessed alongside intraspecific variation among populations of the focal species. We used phylogenetic analyses, multivariate analysis, and best model selection to assess the relative roles of elevation, diversity, and phylogenetic context in shaping thermal strategies. Cold tolerance and leaf area showed strong phylogenetic conservatism across taxa, whereas heat tolerance exhibited little phylogenetic structure. Mid‐elevation assemblages, characterized by peak species richness, showed greater divergence in thermal tolerance among species. In contrast, *P. flammea* populations displayed their broadest thermal tolerance at high elevations, coinciding with high monocot diversity and phylogenetic clustering, as well as harsher abiotic conditions. Lowland populations were the most abundant in assemblages with lower representation of closely related taxa, but exhibited reduced heat tolerance. Overall, community diversity and phylogenetic structure explained variation in thermal strategies better than elevation alone, indicating that biotic context, captured here through patterns of community diversity and phylogenetic relatedness, is an important correlate of thermal niche differentiation along tropical elevational gradients.

## Graphical abstract

We examined how community diversity and phylogenetic context are associated with plant thermal strategies along a tropical elevational gradient in the Brazilian Atlantic Forest, using *Pitcairnia flammea* as a focal species. By integrating community surveys, phylogenetic structure, functional traits, and experimental estimates of heat and cold tolerance, we found that variation in thermal strategies was more strongly associated with biotic context than with elevation alone. These findings suggest that incorporating community composition and evolutionary history can improve understanding of trait differentiation and predictions of species responses to climate change in species‐rich tropical systems.

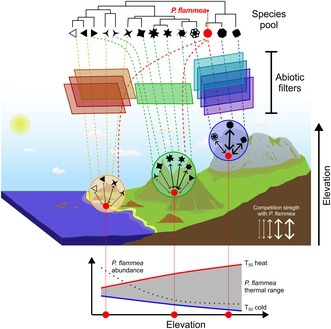

## 1 Introduction

Species are not evenly distributed across the Earth (Antonelli et al. 2023). Increasing seasonality toward higher latitudes and elevations strengthens abiotic filtering, favoring environmental generalists with broad thermal tolerance ranges (von Humboldt 1807; Wallace 1878; Dobzhansky 1950), whereas the milder, more climatically stable conditions of the tropics allow the persistence of narrowly adapted specialists and contribute to the region's exceptional diversity (Fischer 1960; Klopfer and MacArthur 1961; Schemske et al. 2009). The tropical diversity comes through a denser packing of niche space, reducing niche overlap and promoting coexistence (Pigot et al. 2016; Pellissier et al. 2018; Aros‐Mualin et al. 2021; Hughes et al. 2022; de Oliveira et al. 2025). Over evolutionary timescales, biotic interactions can further drive adaptive divergence and displace species' realized niches and range limits away from expectations based on abiotic constraints alone (Darwin 1859; Schluter 2000; Wiens 2011; Wisz et al. 2013; Diamond et al. 2017; Martin and Ghalambor 2023). For broad‐ranging species in particular, spatial variation in community composition may therefore shape population‐level trait divergence across a species' range (Case et al. 2005; Svanbäck and Bolnick 2007; Ricklefs 2010).

Biotic interactions also vary along environmental gradients, as the relative importance of abiotic constraints and community context shifts across space. In benign environments, strong competitors tend to dominate and displace weaker competitors into more stressful conditions, where stress‐tolerant strategies are favored, consistent with the competitive exclusion–tolerance rule (Martin and Ghalambor 2023). Subordinate species may even shift their realized tolerance limits in response to this competitive pressure (Martin and Ghalambor 2023). Negative interactions structure communities most strongly in milder environments (Darwin 1859; MacArthur 1984; Chick et al. 2020; Paquette and Hargreaves 2021), whereas positive interactions such as facilitation become increasingly important toward more stressful conditions, buffering the fitness constraints imposed by abiotic stress (Bertness and Callaway 1994; Maestre et al. 2009).

The direction and strength of these interactions depend jointly on the abiotic context and on the composition of the community (Maestre et al. 2009). Strong abiotic filtering tends to assemble phylogenetically related species that share similar traits and tolerances, reflecting the phylogenetic conservatism of many ecologically relevant traits (Blomberg et al. 2003; Nuismer and Harmon 2015). Such species may initially coexist, but as their relative abundance increases, competition and the eventual exclusion of weaker competitors become more likely (Violle et al. 2011). Relative abundances therefore modulate the intensity and symmetry of interactions (Slatkin 1980; Vázquez et al. 2007; Mönkkönen et al. 2017). In species‐rich tropical systems, where most taxa are locally rare, interactions are likely diffuse and context dependent, involving many species at once rather than discrete pairs (Gross 2008; Aschehoug and Callaway 2015; Ovaskainen et al. 2017). Characterizing how biotic context shapes ecological strategies in tropical systems therefore requires moving beyond pairwise comparisons toward community‐level diversity and phylogenetic structure as joint descriptors of the biotic context in which traits are expressed.

**FIGURE 1:**
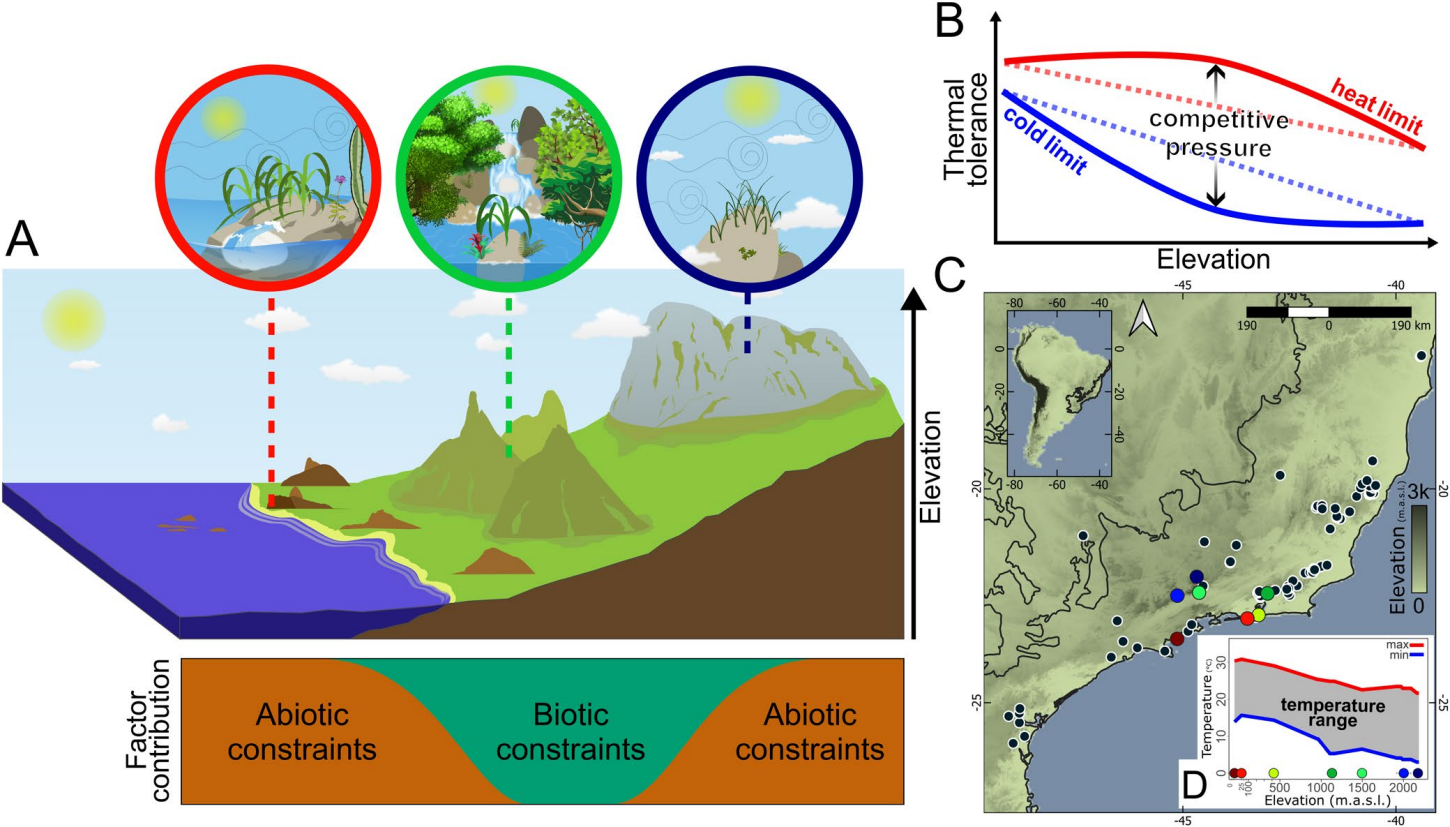
Conceptual framework and a priori predictions for how community diversity relates to plant thermal strategies across a tropical elevational gradient, and for population variation in *Pitcairnia flammea*. (A) At low (coastal; red) and high (montane; blue) elevations, strong abiotic constraints (e.g., thermal extremes, salinity, disturbance; orange region) are expected to dominate community assembly. At mid‐elevations (rainforest; green), where canopy cover and species richness peak, biotic context is expected to become relatively more important (green region). (B) Under a thermal‐tolerance framework, species‐rich assemblages—particularly those with higher diversity of closely related taxa—are expected to be associated with greater divergence in thermal limits and broader tolerance ranges (solid lines) than predicted from abiotic conditions alone (dotted lines). (C) Elevation and geographic distribution of sampled assemblages co‐occurring with *P. flammea* across the Brazilian Atlantic Forest (redish: lowlands; greenish: mid‐elevations; bluish: highlands; black dots: known occurrences). (D) Variation in minimum (blue) and maximum (red) temperatures across elevations for each locality (WorldClim; Fick and Hijmans 2017), with thermal range shown in gray.

Here, we examine how community diversity and phylogenetic structure relate to plant thermal strategies across a tropical elevational gradient, considering both species‐ and population‐level patterns. Our focal species, *Pitcairnia flammea* Lindl., is a rupicolous bromeliad widely distributed in the Brazilian Atlantic Forest (17°–26° S), from coastal rocky shores to elevations above 2200 m a.s.l., where it co‐occurs with mat‐forming monocots (Smith and Downs 1974; de Paula et al. 2016; Porembski et al. 2021). Despite strong genetic structure among its populations, with high‐elevation lineages diverging earliest and showing greater thermal tolerance, a tendency that shifts toward heat‐avoidance at low elevations (Mota et al. 2020; Chaves et al. 2024; Cacossi et al. forthcoming), the thermal strategies of *P. flammea* relate weakly to elevation, suggesting a potential effect of biotic and evolutionary factors (Figure 1A). We address this at two scales, across co‐occurring species and among populations of *P. flammea* , testing whether (i) thermal tolerance and associated leaf traits vary with elevation but diverge most in species‐rich assemblages (Figure 1B); (ii) across co‐occurring species, variation in thermal tolerance is more strongly associated with community diversity and phylogenetic structure than with elevation alone; and (iii) among *P. flammea* populations, thermal strategies covary with the diversity and composition of co‐occurring plant groups, especially closely related taxa, such that elevation becomes a weaker predictor of population‐level thermal tolerance where community diversity is high.

## 2 Material and Methods

### 2.1 Sampling Design

To characterize the plant assemblages co‐occurring with *P. flammea* across elevations, we surveyed seven localities spanning the species' elevational distribution (see Figure 1C). At each locality, we established three 5 × 5 m plots, centered on randomly selected *P. flammea* individuals, and within each plot, we randomly placed three 1 m^2^ quadrats. For every plant species ≥ 5 cm in height, we recorded presence and estimated percentage cover (adapted from Munhoz and Araújo 2011). We adopted the absolute rather than relative cover because life‐forms may overlap vertically. Taxonomic classification followed the APG IV system (2016) for angiosperms and the PPG I system (2016) for ferns or lycophytes. For each plot, light availability was characterized by measuring canopy cover (CC; ratio of shadow to total area in px^2^; Chianucci 2015) from hemispherical photographs taken with a 7.5 mm focal length fish‐eye lens (238° angle; Liginn 238) at a height of ~1.3 m above ground levels. To assess the effect of co‐occurring taxa differing in relatedness to *P. flammea* , we assigned species to three groups: dicots, monocots, and ferns (including lycophytes).

### 2.2 Diversity and Phylogenetic Structure

For each assemblage, we quantified species diversity as species richness and the Shannon index, using species cover as an abundance proxy (most species are herbaceous and largely clonal, making the discrete individuals unreliable to define). Diversity was computed for the whole assemblage and for each group, using the “vegan” and “picante” R packages (Kembel et al. 2010; Oksanen et al. 2024). To assess phylogenetic structure, we estimated the standardized effect size of the mean nearest taxon distance (SES‐MNTD; Webb et al. 2008; Keck and Kahlert 2019), which measures how closely related co‐occurring species are relative to a null expectation. We used a “richness” null model, randomizing species abundances within samples while preserving richness, applied to a filtered presence–absence matrix and the cophenetic distances from the pruned phylogeny. Positive and negative SES‐MNTD values indicate, respectively, that co‐occurring species are less or more closely related than expected by chance (i.e., phylogenetic overdispersion or clustering). Phylogenetic relationships were reconstructed with “V.PhyloMaker” (Jin and Qian 2019), based on the “PhytoPhylo megaphylogeny” (Zanne et al. 2014; revised by Qian and Jin 2016; 31,389 species), pruned to match our species list. For analysis combining diversity with functional traits (below), we used the Shannon index only.

### 2.3 Thermal Tolerance and Functional Traits

To select the species characterized functionally and thermally at each locality, we used importance values (IV), calculated per species as the sum of its cover and frequency relative to all plants in each subplot (RCi + RFi). This prioritizes the dominant species, taken as proxies for the interaction environment experienced by *P. flammea* . At each locality, we sampled five to seven species (including *P. flammea* ), with three to six individuals each depending on local abundance; when *P. flammea* did not rank among the top five IV species, it was added, and if those five comprised fewer than five distinct genets, the next‐ranked species was included. For *P. flammea* specifically, traits and thermal tolerance were estimated from five individuals per population, allowing population‐level variation to be assessed independently of community‐level patterns.

For each sampled individual we measured photosynthetic heat and cold tolerance and several leaf traits: leaf mass per area (LMA; dry weight/area), succulence index (SI; Ogburn and Edwards 2010), leaf area (LA; “Petiole Pro” app, v23.11.8), and stomatal density on the abaxial (StomABA) and adaxial (StomADA) surfaces, summed as total density (StomTOT). For stomatal density, we collected mid‐leaf fragments in neutral buffered formalin (Schneider and Clark 1981), vacuum‐treated to remove air and dehydrated in an ethanol series (to 70%) for storage. Epidermis was dissociated from 1 cm^2^ mid‐leaf fragments (apex, base, and one margin removed in smaller leaves) with a 1:1 hydrogen peroxide–glacial acetic acid solution at 60°C for 12 h, stained with 1% aqueous safranin, and mounted in 50% glycerol (modified from Franklin 1945). Using an Olympus BX51 microscope with a DP71 camera, we imaged 1 mm^2^ intercostal regions at 10× and counted stomata in ImageJ (Schneider et al. 2012).

Heat and cold tolerance were measured shortly after noon and just after sunrise, capturing the warmest and coolest periods and accounting for daily shifts in tolerance (e.g., Chaves et al. 2018). From the youngest fully expanded leaf of each individual we cut ~1.5 cm^2^ discs, dark‐adapted them for 10 min at ~25°C, and measured the potential quantum efficiency of PSII (*F*_v_/*F*_m_) with a fluorometer (PAR‐FluorPen FP 110/D, Photon Systems Instruments); thin or small leaves were grouped to reach the required area for the fluorometer. Samples were sealed in plastic bags and ramped in thermal baths: for heat tolerance, from 27°C to 60°C at 1°C every 3 min, with *F*_v_/*F*_m_ read every 3°C; for cold tolerance, from 20°C to −22°C at a comparable rate, with *F*_v_/*F*_m_ read every 5°C. Samples stabilized ~1 min at each target temperature before reading, with at least 10 min between measurements; leaf temperatures were verified with K‐type thermocouples (TH‐096, Instrutherm) on a subset. Heat and cold tolerance (T_50_heat, T_50_cold) were estimated as the temperatures at which *F*_v_/*F*_m_ dropped to 50% of its room‐temperature value, by fitting sigmoid curves with the “drc” R package (Ritz et al. 2015), following Knight and Ackerly (2003), Gimeno et al. (2009), and Godoy et al. (2011).

### 2.4 Statistical Analysis

To describe variation along the gradient, we regressed each diversity index and species cover on elevation using second‐degree polynomial models, fitting the whole assemblage and each plant group (monocots, dicots, and ferns) separately, with *P. flammea* split from the other monocots. As a sensitivity check, we also fitted generalized additive models (GAMs) with thin‐plate regression splines (“mgcv”; Wood et al. 2016). Polynomial and GAM fits were comparable (AICc), so we retained the polynomial models for their greater interpretability.

We quantified the phylogenetic signal of thermal tolerance and the other functional traits with Pagel's *λ* (Pagel 1999; “phytools”, Revell 2024), computed on species mean values standardized after capping outliers at the 5th and 95th percentiles. *λ* ranges from 0 (no signal) to 1 (Brownian‐motion expectation); significance was assessed by likelihood‐ratio tests with 1000 randomisations.

To reduce the set of correlated traits and test whether trait axes related more to elevation or to diversity, we ran a principal component analysis (PCA), imputing missing trait values with a regularized iterative algorithm on the first two components (“missMDA”; Josse and Husson 2016). Complete‐case and imputed analyses gave nearly identical variance partitioning and loadings. We then modeled the leading components against elevation, assemblage‐level Shannon diversity, or group‐level Shannon diversity using second‐degree polynomials, comparing models by AIC and reporting *F*, *R* ^2^, and *p* (95% CI).

To test whether heat and cold tolerance were better predicted by elevation, plant group, or diversity (Shannon at assemblage and group levels), we ran two parallel pipelines. For all species except *P. flammea* , we fitted phylogenetic generalized linear mixed models (pGLMMs; “phyr”, Ives et al. 2020), testing every predictor combination (including null and full models), with random intercepts for species and individuals and Pagel's *λ* estimated jointly. We excluded *P. flammea* here because its presence in every assemblage would bias relatedness estimates. Models and the phylogenetic term were compared by AIC and likelihood‐ratio tests (*α* = 0.05). For *P. flammea* populations, we fitted second‐degree polynomial regressions of thermal tolerance on elevation and on the Shannon diversity of dicots, monocots, ferns, and the whole assemblage, in all combinations (plus null models). To avoid collinearity, we dropped one variable from any pair with |*r*| > 0.5, keeping the higher univariate *R* ^2^. In both pipelines, best models were those with ΔAIC < 2 and the fewest parameters.

## 3 Results

### 3.1 Diversity and Phylogenetic Structure Along the Elevational Gradient

**FIGURE 2:**
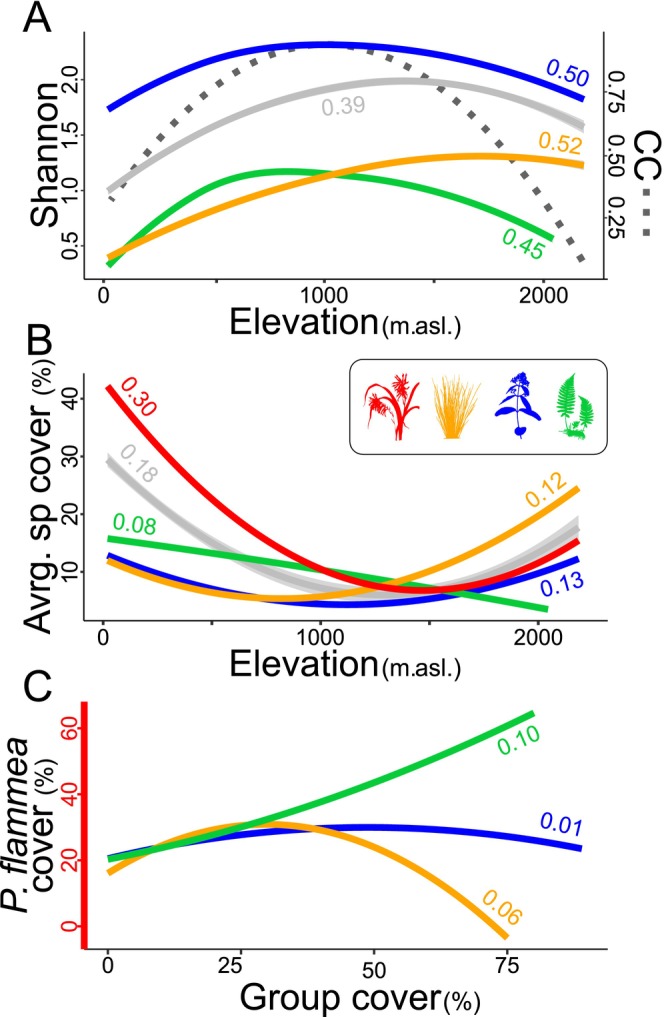
Variation in species composition along the analyzed elevational gradient and their relationships with the cover of the analyzed groups (blue: dicots, orange: monocots, green: ferns, red: *P. flammea* populations, gray: overall species). (A) Changes in species diversity (Shannon index, full lines) and canopy cover (CC, dotted lines). (B) Average species cover of each group along the elevation gradient. (C) The relationship between the total cover of *P. flammea* populations and the combined species cover of the other groups across the elevational gradient. The numbers indicate *R* ^2^ values. All relations showed were significant (*p* < 0.05).

We recorded 209 plant species across all sampled localities. Community‐level Shannon diversity and species richness exhibited a hump‐shaped pattern along the elevational gradient, peaking at mid‐elevations (Shannon up to 3.1; richness up to 78 species) and coinciding with the highest values of canopy cover (CC; up to ~0.9; Figure 2A; Figure S1). This unimodal pattern is held under both polynomial and GAM formulations. Average species cover, by contrast, declined at these elevations (Figure 2B). Phylogenetic structure varied systematically with elevation: higher‐elevation assemblages showed significantly lower SES‐MNTD (down to −2.3), indicating stronger phylogenetic clustering across monocots, dicots, and ferns (Figure S1).

When analyzed by major plant groups, diversity and cover patterns diverged markedly from the community‐level trends. Monocot diversity and average cover increased steadily with elevation, whereas dicot diversity and cover mirrored the overall hump‐shaped pattern. Fern cover declined sharply at the highest elevations (Figure 2A,B). *P. flammea* showed highest cover in lowland sites, with abundance declining as monocot cover increased along the gradient (Figure 2B). Notably, *P. flammea* cover declined strongly when total monocot cover exceeded ~40% of subplot area, whereas its relationship with fern cover was positive and nearly linear (Figure 2C).

### 3.2 Thermal Tolerance Variation Across Species and Elevations

**FIGURE 3:**
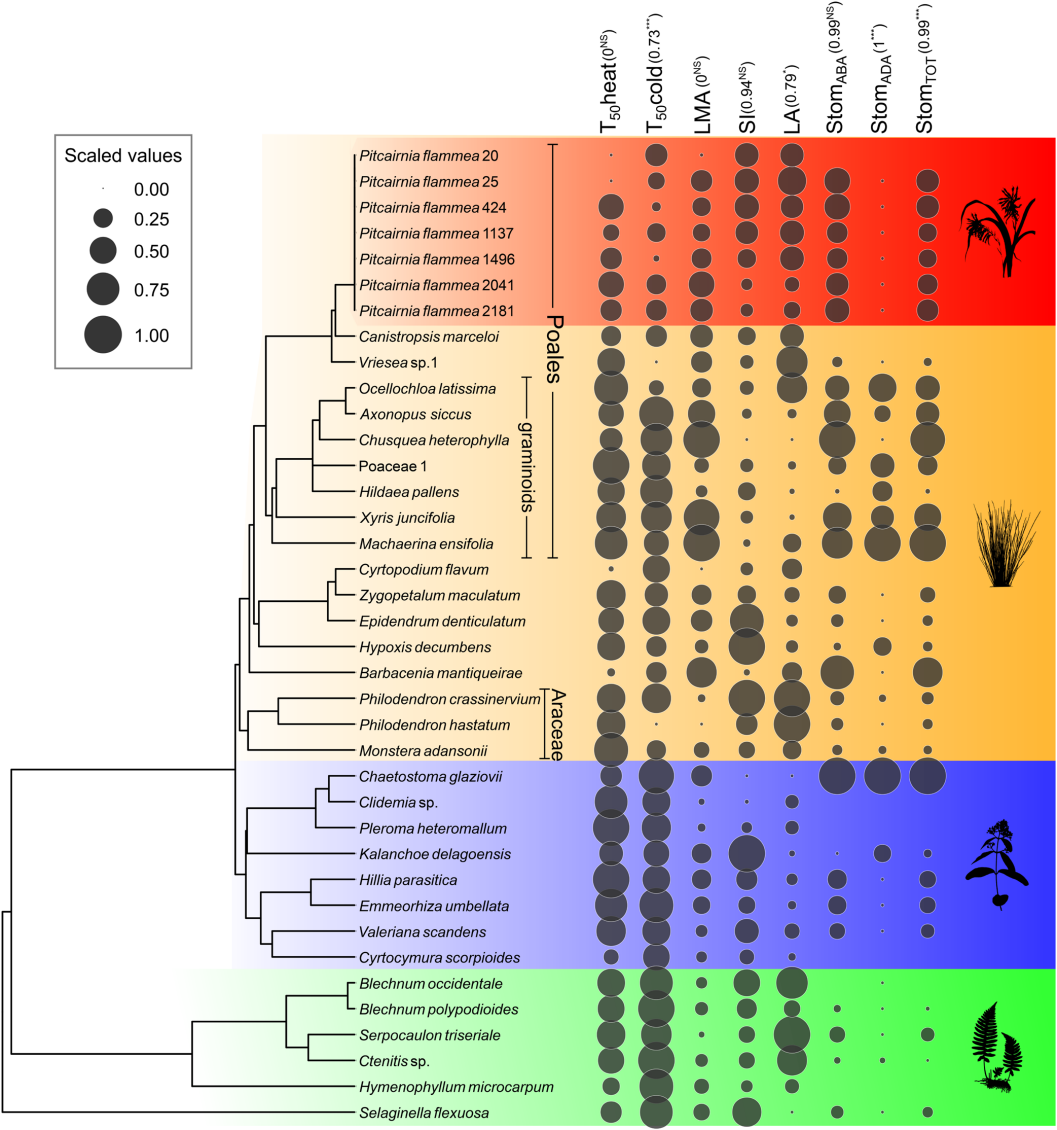
Phylogenetic tree of all species sampled for the estimation of thermal tolerance and functional traits in all plant groups (red: *P. flammea* populations, orange: monocots, blue: dicots, green: ferns). The numbers in front *P. flammea* populations indicate their average elevations (in m.a.s.l.). The scaled values (ranging from 0 to 1) of the average measurements for each trait are represented by circles, with their size corresponding to the values in the columns for each trait. The phylogenetic signal (Pagel's *λ*) and its statistical significance for each trait are indicated in parentheses following the trait name. Traits include T_50_heat (heat tolerance), and T_50_cold (cold tolerance), LMA (leaf dry mass per area), SI (succulence index), LA (leaf area), Stom_ABA_ (stomata density on the abaxial leaf surface), Stom_ADA_ (stomata density on the adaxial leaf surface), and Stom_TOT_ (total stomata density on both leaf surfaces). Statistical significance is denoted as NS (*p* > 0.05), *p < 0.05, **p < 0.01, and ***p < 0.001.

Across all groups, variation in cold tolerance (T_50_cold) consistently exceeded variation in heat tolerance (T_50_heat; Figure S2), and this contrast increased with elevation: lowland assemblages exhibited similar variability in both, whereas high‐elevation assemblages showed cold tolerance variability more than five times greater than that of heat tolerance (Figure S2). T_50_ heat values ranged from 43.3°C (lowland *P. flammea* ) to 58.3°C (mid‐elevation Poaceae), while T_50_cold values ranged from 13.0°C (mid‐elevation *Ctenitis* sp.) to −61.7°C (mid‐elevation *Philodendron hastatum*). These extreme values reflect fitted sigmoidal responses of PSII responses and were retained in all analyses; sensitivity analyses excluding extreme observations yielded qualitatively similar patterns (Figure S2). Cold tolerance showed a strong phylogenetic signal across species (Pagel's *λ* = 0.73, *p* < 0.001), whereas heat tolerance showed none (*λ* ≈ 0; Figure 3). Mid‐elevation populations of *P. flammea* ranked among the most cold‐tolerant taxa sampled (−37.9°C and −36.7°C), exceeded only by *P. hastatum* and a mid‐elevation bromeliad (*Vriesea* sp., −42.7°C), while lowland *P. flammea* populations exhibited the two lowest heat tolerance values observed (43.3°C and 44.1°C).

### 3.3 Thermal Tolerance: Elevation Versus Biotic Context

**FIGURE 4:**
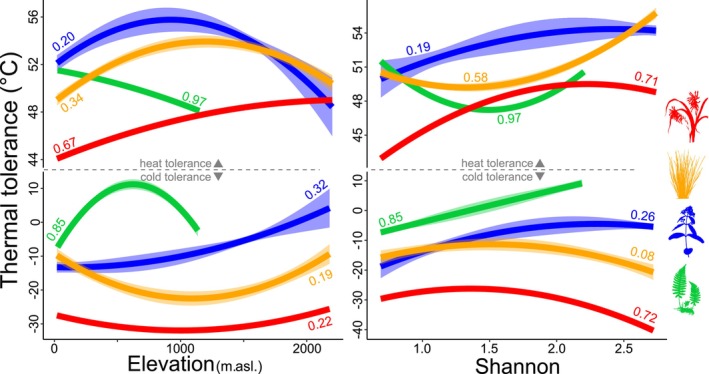
Relationship between thermal tolerance and both elevation and species diversity (Shannon Index). The plots show all significant second‐degree polynomial functions for each group (*p* < 0.05): blue for dicots, orange for monocots, green for ferns, red for *P. flammea* . *R* ^2^ values are displayed for each relationship, indicating the strength of the fit between thermal tolerance and the variables.

Thermal tolerance breadth of most plant groups followed an unimodal pattern along the elevational gradient, with narrower thermal ranges at low and high elevations and broader ranges at mid‐elevations (Figure 4A). In contrast, *P. flammea* showed its widest thermal tolerance breadth at high elevations, driven primarily by increased heat tolerance rather than large shifts in cold tolerance (Figure 4A). When examined along the diversity gradient, distinct patterns emerged among plant groups. In dicots, increasing community diversity was associated with shifts toward higher T_50_ values for both heat and cold tolerance, whereas monocots showed an overall expansion of thermal tolerance breadth with increasing diversity (Figure 4B). Although *P. flammea* followed the general monocot trend, it declined particularly steeply in heat tolerance in species‐poor assemblages (Figure 4B).

When *P. flammea* was excluded, species‐level phylogenetic generalized linear mixed models (PGLMM) that included both elevation and diversity metrics showed better fit than models with elevation alone for both heat and cold tolerance (Figure S4B,C). Cold tolerance models retained a significant phylogenetic signal (*λ* = 0.53, *p* < 0.001), whereas heat tolerance showed negligible phylogenetic structure (*λ* < 0.01; Figure S4B,C). Among *P. flammea* populations, thermal tolerance covaried strongly with community composition: heat tolerance was best predicted by monocot diversity (*R* ^2^ = 0.38, *p* < 0.001), while cold tolerance was best explained by a combination of overall and dicot diversity (*R* ^2^ = 0.61, *p* < 0.001; Figure 6; Figure S4D,E). Higher diversity across plant groups was associated with broader thermal‐tolerance breadth in *P. flammea* (Figure 6B).

### 3.4 Functional Trait Variation and Multivariate Structure

Functional traits showed consistent elevational trends across groups: lowland plants had higher succulence, lower LMA, larger leaves, and lower stomatal density, with the opposite at high elevations (Figure S3). In *P. flammea* , mean leaf area declined sharply from 135 cm^2^ in lowland populations to 40.5 cm^2^ at high elevations. Leaf area showed the strongest phylogenetic signal among all measured traits (*λ* = 0.79, *p* = 0.04; Figure 3). Within monocots, graminoid species—except for the broad‐leaved *Ocellochloa latissima* —had markedly smaller leaves (mean = 6.7 cm^2^) than bromeliads (107.7 cm^2^) and aroids (262.8 cm^2^), consistent with their dominance at higher elevations. Stomatal traits showed more heterogeneous patterns. Although abaxial stomatal density increased with elevation across most groups (from 9–144.5 to 51.5–425 stomata mm^−2^), variation within *P. flammea* was comparatively weak, with mid‐elevation populations exhibiting both the highest (198.4) and lowest (55 stomata mm^−2^) values (Figure S3). About 60% of the analyzed species lacked adaxial stomata, including *P. flammea* , whereas the highest adaxial stomatal density was recorded in the highland monocot *M. ensifolia* (241 stomata mm^−2^).

**FIGURE 5:**
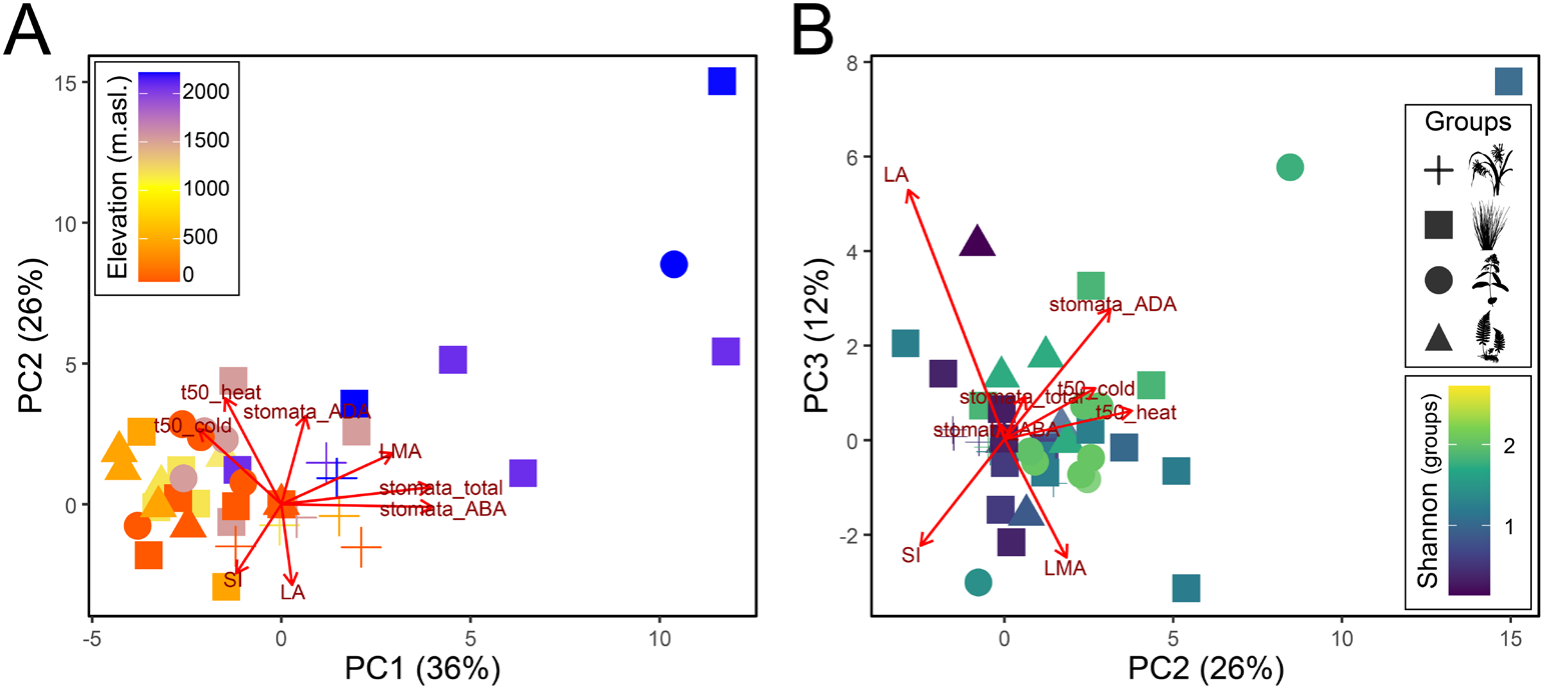
Principal Component Analysis (PCA) of species traits. Ordination of plots along the first three principal components (PC1–PC3), with points colored by elevation (A) or Shannon diversity of each plant group (B). Color gradients reflect the variables best explaining variation along the axes: Elevation primarily explains variation along PC1, while Shannon diversity best explains PC2 and PC3, as supported by AIC‐based model comparisons. Point shapes indicate plant groups ( *P. flammea* populations, monocots, dicots, and ferns; see legend). Red arrows represent trait loadings, highlighting the contribution of each trait to PCA axes.

Principal component analysis captured 64% of total variance across the first three axes (Figure 5). PC1, associated mainly with stomatal traits and LMA, was best explained by elevation (*F* = 165, *R* ^2^ = 0.11, *p* < 0.001), whereas PC2 and PC3, driven largely by thermal tolerance and leaf area, were best explained by group‐level Shannon diversity (PC2: *F* = 781, *R* ^2^ = 0.36; PC3: *F* = 117, *R* ^2^ = 0.08; both *p* < 0.001; Figure S4A). Complete‐case (*n* = 1633 individuals) and missMDA‐imputed PCAs gave nearly identical variance partitioning (PC1: 0.37 vs. 0.36; PC2: 0.24 vs. 0.26) and highly concordant loadings (Pearson's *r* = 0.994 for PC1, 0.992 for PC2), indicating the trait structure was robust to missing‐data treatment.

## 4 Discussion

**FIGURE 6:**
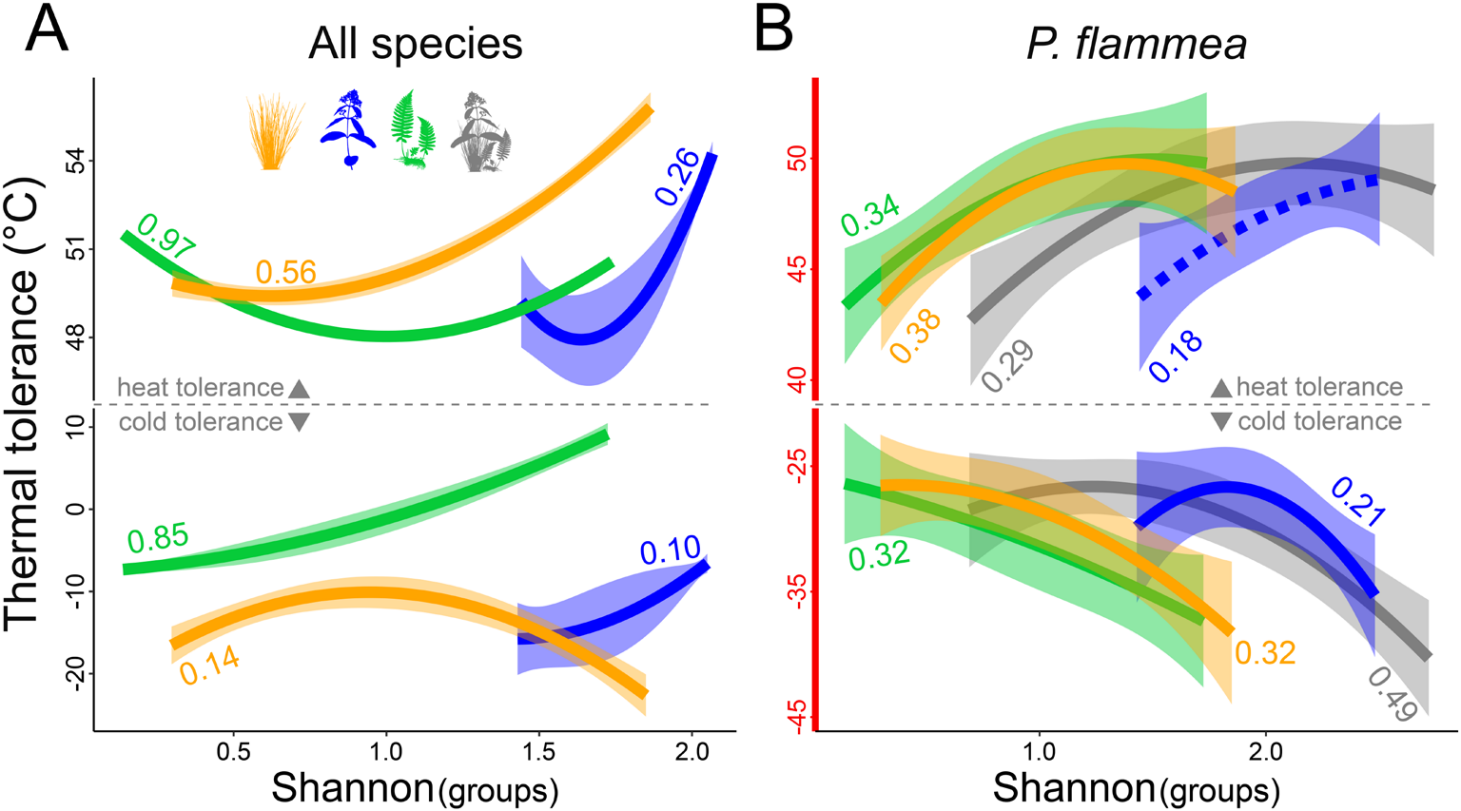
Relationship between thermal tolerance and Shannon diversity of each plant group. Colors represent plant groups: orange = monocots, blue = dicots, green = ferns, and gray = whole assemblage. Panel (A) shows second‐degree polynomial regressions between the thermal tolerance of all species except *P. flammea* and the Shannon diversity of each respective group. Panel (B) illustrates the relationships between the thermal tolerance of *P. flammea* populations and the Shannon diversity of each group, as well as the whole assemblage. *R* ^2^ values are shown for each model, indicating the strength of the relationship. Dotted lines represent non‐significant relationships (*p* > 0.05).

Our results indicate that plant thermal strategies across this tropical elevational gradient are associated with both abiotic conditions and community context. Consistent with our predictions, thermal traits varied along the gradient but diverged most in species‐rich assemblages, and at both scales examined, community diversity and phylogenetic structure, particularly within phylogenetically proximate groups, were more strongly associated with thermal tolerance than elevation alone (Figures 4 and 6). Cold tolerance and leaf area remained phylogenetically conserved across taxa, whereas heat tolerance showed little phylogenetic structure (Figure 3). Among *Pitcairnia flammea* populations, thermal strategies covaried with the diversity of co‐occurring plant groups, such that elevation became a weaker predictor of population‐level thermal tolerance where diversity was high (Figure 6B). Together, these patterns indicate that in species‐rich tropical systems, community diversity and phylogenetic context are integral to thermal niche differentiation and should be considered when interpreting elevational patterns and forecasting responses to environmental change.

### 4.1 Species Composition and Thermal Tolerance Patterns

Species in more diverse or phylogenetically clustered assemblages exhibited broader thermal niche breadths (Figures 4B and 6A), a pattern more strongly associated with the diversity of closely related taxa than with elevation alone. This was evident both across species and within *Pitcairnia flammea*: among its populations, heat tolerance was most strongly associated with monocot diversity, and cold tolerance with overall and dicot diversity (Figure 6B). These patterns are consistent with a shift in the dominant assembly process along the gradient, in line with the stress‐dominance hypothesis, whereby abiotic filtering gains importance and density‐dependent interactions decline toward more stressful conditions (Grime 1977; Swenson and Enquist 2009). Thermal seasonality is a central axis of this filtering, selecting for wider thermal‐tolerance breadth in more seasonal environments and narrower breadth in stable ones (Janzen 1967; Ghalambor et al. 2006; Sklenář et al. 2023), such that the relative roles of abiotic filtering and biotic context shift systematically across the three elevational zones we sampled.

Across these elevational zones, phylogenetic structure and thermal tolerance patterns tracked the shifting balance between abiotic filtering and biotic pressures. At high, seasonal elevations, filtering selects for closely related, thermally similar lineages, producing phylogenetic clustering (negative SES‐MNTD; Figure S1) and a diverse graminoid flora (Poaceae, Cyperaceae, and Xyridaceae; Figure 2A,B) with reduced leaf area (Givnish et al. 2011; Miazaki et al. 2015). Consistent with this, Poales richness, the order of *P. flammea* , increased markedly above 1000 m, and highland *P. flammea* populations reached their highest heat tolerance (Figure 4A) where monocot diversity and clustering peaked; as these relatives accumulate, the biotic context intensifies (Figure 2C) even as overall richness remains constrained by strong abiotic limits (Hargreaves et al. 2014; Paquette and Hargreaves 2021). Mid‐elevations, milder and most diverse, showed reduced dominance by any single taxon and the greatest divergence in thermal tolerance among co‐occurring species (Figure 4A). At low elevations, low thermal seasonality coincided with the narrowest thermal ranges (Figure 4A), consistent with a reliance on heat‐avoidance over physiological tolerance (Chaves et al. 2024, 2026); there, assemblages were more distantly related to *P. flammea* , with higher fern cover, likely relaxing biotic pressure and allowing its highest abundance (Figure 2B,C), consistent with the weaker genetic structure among lowland populations (Cacossi et al. forthcoming). Across these zones, variation in thermal strategies tracked species composition and phylogenetic structure rather than elevation alone, with abiotic seasonality and stress setting the template within which thermal strategies are expressed.

### 4.2 Phylogenetic Conservatism of Cold Tolerance and Leaf Area

The exceptional cold tolerance of *Pitcairnia flammea* across assemblages is consistent with the evolutionary history of Bromeliaceae. Although the biogeographic origin of *Pitcairnia* remains debated (Givnish et al. 2007, 2011; Schütz et al. 2016), the strong phylogenetic signal in cold tolerance (Figure 3) mirrors patterns in other clades (Lancaster and Humphreys 2020; Nie et al. 2023), indicating deep evolutionary constraints, whereas the comparatively low cold tolerance of ferns and lycophytes likely reflects diversification under warm climates (Lehtonen et al. 2017; Barrera‐Redondo et al. 2018). Within *P. flammea* , this deep history is also apparent among populations: high‐elevation lineages diverged earliest and persist longest along the gradient (Cacossi et al. forthcoming), consistent with the smoother variation in cold tolerance we recovered across *P. flammea* populations relative to the broader plant groups. Common‐garden experiments on these same populations clarify this contrast: heat tolerance and its avoidance traits were strongly canalized under uniform conditions, whereas cold tolerance was highly plastic, declining by up to ~18°C (Chaves et al. 2026). The weak phylogenetic structure of heat tolerance therefore reflects a canalized, locally adapted trait, while the strong cold‐tolerance signal coexists with high within‐species plasticity.

Leaf area likewise exhibited strong phylogenetic conservatism across clades (Figure 3; *λ* = 0.79). Dicots generally displayed smaller leaves and lower cold tolerance, whereas graminoid monocots followed a similar trend—except for *Ocellochloa latissima* , whose broad, cold‐tolerant leaves resembled those of bromeliads, including *P. flammea* . Reduced leaf area in graminoids, the most diverse clade in highland sites, and in other cold‐adapted species (e.g., *Chaetostoma glaziovii*) is consistent with strategies that limit exposure to cold‐induced stress (Midolo et al. 2019; Liu et al. 2020). Broad‐leaved lineages, by contrast, likely require compensatory physiological adjustments to persist under cold conditions (Chaves et al. 2024). Within *P. flammea* , common‐garden results further indicate that part of the among‐population variation in leaf area is environmentally masked in the field and expressed under favorable conditions, consistent with phenotypic plasticity and the release of cryptic genetic variation (Schlichting 2008; Archambeau et al. 2023; Chaves et al. 2026). This coexistence of macroevolutionary constraint and context‐dependent expression provides a plausible mechanism for the broad elevational range of *P. flammea* .

### 4.3 Elevational Patterns in LMA and Leaf Succulence

Distinct from thermal tolerance, other functional traits exhibited contrasting elevational patterns across assemblages and among *Pitcairnia flammea* populations. At low elevations, plants were characterized by higher leaf succulence and lower leaf mass per area (LMA; Figure 5A; Figure S3), traits associated with water storage and the maintenance of physiological function under hot and dry conditions (Midolo et al. 2019; Lim et al. 2020; Griffiths and Males 2017; Males 2017; Chaves et al. 2024). These traits involve trade‐offs, since succulent tissues generally show lower freezing resistance, constraining persistence under colder, higher elevations (Nobel 1982; Von Willert 1992; Grace 2019; Lim et al. 2020). In contrast, high elevations were associated with higher LMA, particularly among monocots (Figure 5A), reflecting greater structural investment and nutrient retention under chronically stressful environments (Diemer 1998; Westoby et al. 2002; Wright et al. 2002, 2005). Dicots showed a weaker LMA–elevation relationship, consistent with the life‐history strategies of many of these species, which reduce exposure to unfavorable seasons through deciduousness or rapid life cycles (Wright et al. 2005; Benavides et al. 2021; Butrim and Royer 2020). Rather than varying independently, these elevational shifts in LMA and leaf succulence define trait combinations (Figure 5) that, together with the seasonality‐driven tolerance gradient, underlie how plants balance tolerance, avoidance, and a likely phenological escape across thermal gradients (May et al. 1962; Levitt 1972; Kooyers 2015).

### 4.4 Biotic Interactions and Historical Assembly of Diversity in Old Tropical Mountains

The southeastern Brazilian Atlantic Forest highlands form an ancient, geologically stable montane system whose history contrasts with that of young, tectonically active ranges such as the Andes. Here, high plant diversity reflects long‐term climatic oscillations rather than recent uplift, favoring dense assemblages of closely related monocots (Figure 2A,B; Figure S1, including bromeliads, grasses, sedges, and Xyridaceae (Hughes and Eastwood 2006; Vasconcelos et al. 2020; Zappi et al. 2017)). Recurrent Pleistocene fluctuations likely drove repeated reshuffling of distributions, increasing lineage co‐occurrence and, combined with strong environmental filtering, intensifying biotic interactions over evolutionary timescales (Cahill Jr. et al. 2008; Vasconcelos et al. 2020). This deep history is also legible within *P. flammea* , whose high‐elevation populations diverged earliest (Cacossi et al. forthcoming), consistent with long‐term persistence in these stable highland environments. Such evolutionary depth helps explain why thermal strategies are expressed within dense assemblages of functionally similar, closely related taxa at high elevations, rather than as a direct response to elevation per se (Figure 5B).

## 5 Conclusion

Thermal niche differentiation in these tropical montane systems reflects not only abiotic gradients but also the historical and biotic contexts in which communities assemble. In old, climatically stable mountains, long‐term climatic oscillations and the resulting accumulation of closely related, functionally similar lineages appear to shape both community structure and the thermal strategies expressed by co‐occurring taxa. Because we did not measure interactions directly, these associations should be read as the biotic and evolutionary context in which thermal strategies are expressed rather than as evidence that interactions drive divergence, since the same phylogenetic patterns are also consistent with abiotic filtering. Even so, the consistent associations among thermal strategies, community diversity, and phylogenetic structure (Figures 4, 5, 6), together with common‐garden evidence of canalized heat and plastic cold tolerance in the same populations (Chaves et al. 2026), indicate that biotic and evolutionary context is integral to how these strategies are expressed. As climate change drives upslope range shifts and novel species encounters, the reorganization of community composition is expected to reshape selective contexts in ways that temperature‐based models alone cannot capture, underscoring the need to integrate biotic and phylogenetic context into predictions of tropical montane biodiversity responses.

## Author Contributions

**Cleber J. N. Chaves:** conceptualization (lead), data curation (lead), formal analysis (lead), funding acquisition (equal), investigation (lead), methodology (lead), project administration (lead), visualization (lead), writing – original draft (lead), writing – review and editing (lead). **Marília M. Tavares:** investigation (supporting), methodology (supporting), writing – review and editing (supporting). **Gabriel P. Sabino:** investigation (equal), methodology (equal), writing – review and editing (equal). **João Pedro S. P. Bento:** conceptualization (equal), investigation (supporting), methodology (equal), writing – review and editing (supporting). **Vitor de A. Kamimura:** conceptualization (equal), investigation (equal), methodology (supporting), writing – review and editing (equal). **Wagner L. dos Santos:** methodology (supporting), writing – review and editing (supporting). **Lucas N. Gonçalves:** methodology (equal), writing – review and editing (supporting). **Karina T. Silva:** methodology (supporting). **Juliana L. S. Mayer:** funding acquisition (supporting), resources (equal), writing – review and editing (supporting). **Kenneth J. Feeley:** conceptualization (equal), data curation (equal), investigation (supporting), project administration (equal), resources (equal), supervision (equal), validation (equal), visualization (equal), writing – review and editing (equal). **Clarisse Palma‐Silva:** conceptualization (lead), funding acquisition (lead), project administration (supporting), resources (lead), supervision (lead), writing – review and editing (equal).

## Funding

This work was supported by the Fundação de Amparo à Pesquisa do Estado de São Paulo (FAPESP; grants 2020/14805‐4 and 2024/08569‐7 to C.J.N.C., 2022/09041‐0 to V.A.K., and 2022/07480‐7, 2021/10639‐5, and 2024/01417‐7 to C.P.‐S.) and by the Conselho Nacional de Desenvolvimento Científico e Tecnológico (CNPq; grant 302962/2022 to C.P.‐S.).

## Conflicts of Interest

The authors declare no conflicts of interest.

## Supporting information


**Figures S1–S4:** ece374025‐sup‐0001‐Figures.pdf.

## Data Availability

The data that support the findings of this study are openly available in the Zenodo repository at https://doi.org/10.5281/zenodo.16105564.
