# Supplementary material for: Diversity and Community Structure Underlie Divergence in Thermal Strategies Across Tropical Elevations
Source: Ecol Evol. 2026 Jul 25;16(7):e74025. doi: 10.1002/ece3.74025 (PMC13401213; doi:10.1002/ece3.74025)
Supplement: Supplementary file 1 — Figures S1–S4: ece374025‐sup‐0001‐Figures.pdf. [file ECE3-16-e74025-s001.pdf]

Diversity and community structure underlie divergence in thermal strategies across tropical elevations

Supplementary materials

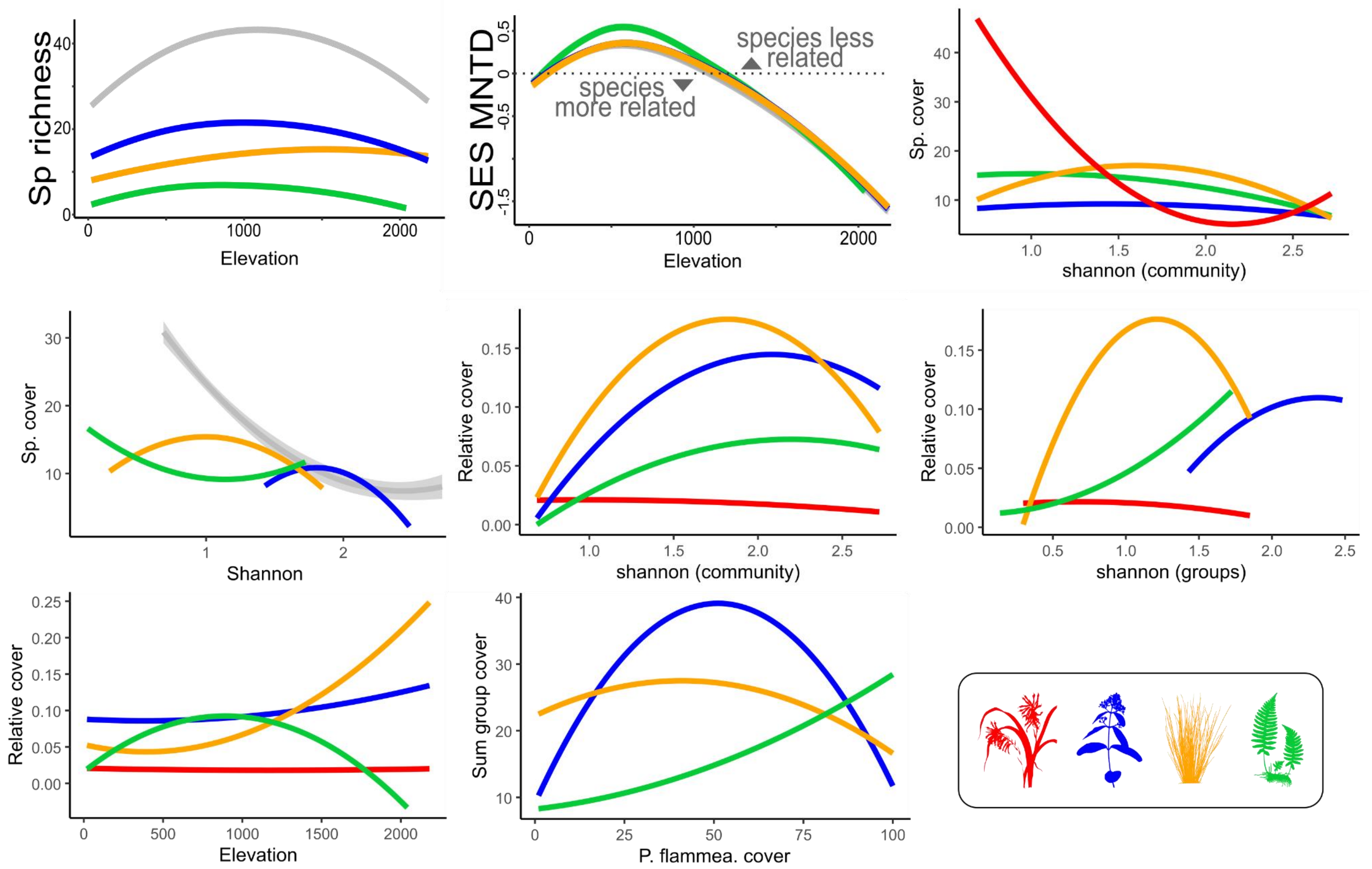

Figure S1. Variation in species composition and cover along the analyzed elevational and diversity gradients according to the analyzed groups (*blue*: dicots, *orange*: monocots, *green*: ferns, *red*: *P. flammea* populations, *gray*: overall species).

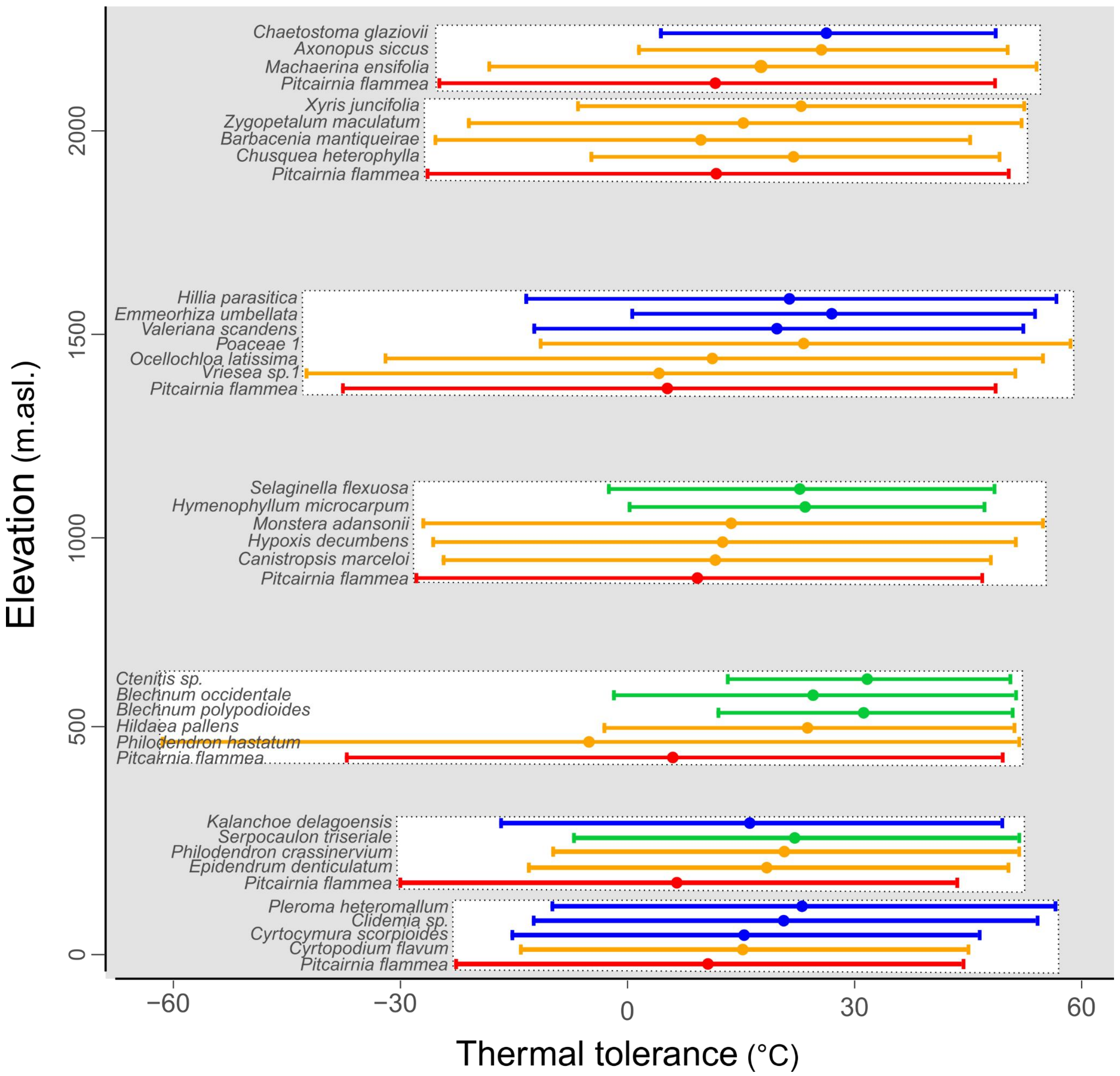

Figure S2. Thermal tolerance ranges (limited by the T_{50}heat on the right and by the T_{50}cold on the left) of all analyzed species across the elevations. Circles indicate the mid-temperature between the heat and cold tolerances. White rectangles circumscribe species from the same locations. *blue*: dicots, *orange*: monocots, *green*: ferns, *red*: *P. flammea* populations.

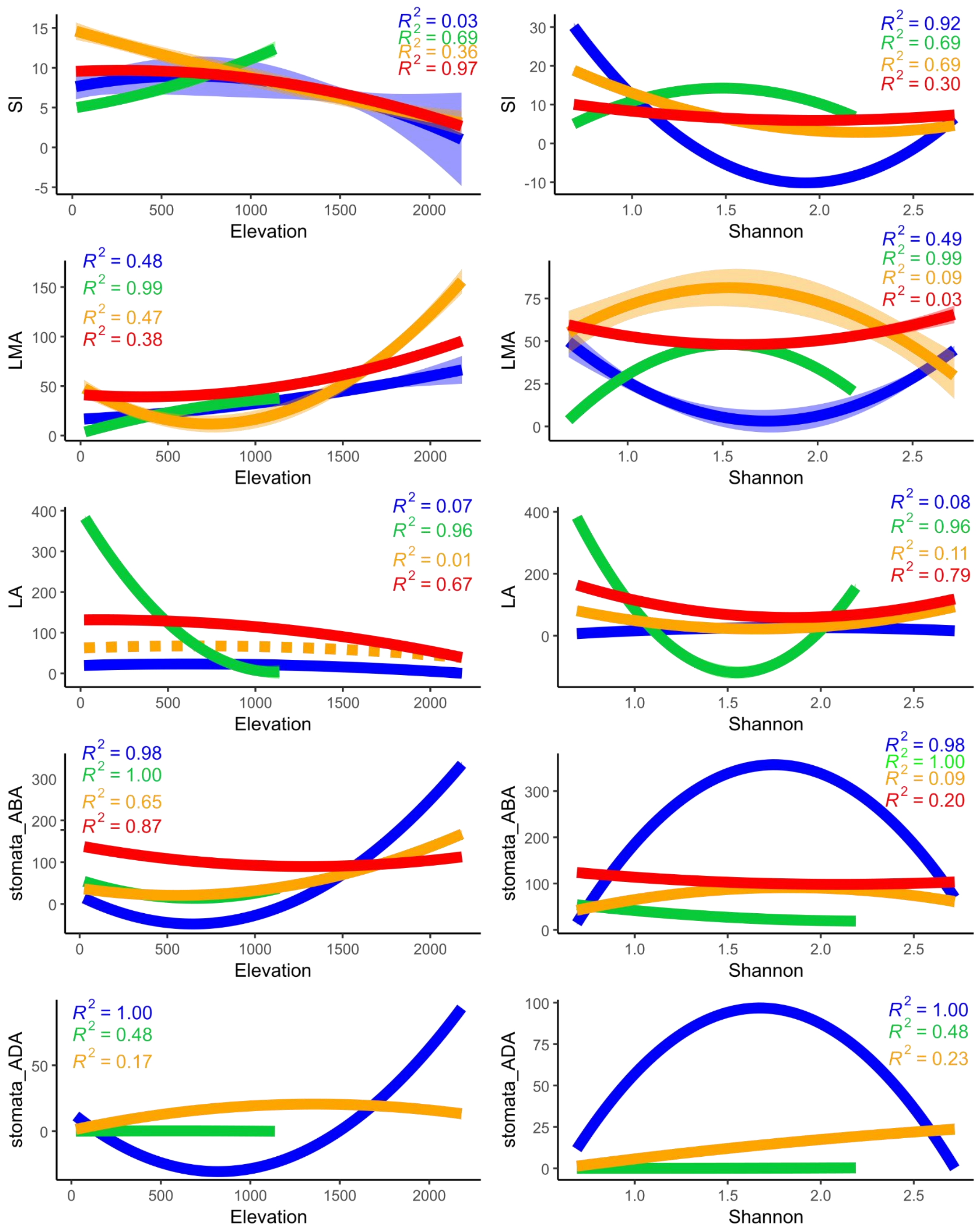

Figure S3. Relationship between all traits measured with both elevation (left column) and species diversity (Shannon Index; right column). The plots show all significant second-degree polynomial functions for each group ($p < 0.05$): blue for dicots, orange for monocots, green for ferns, red for *P. flammea*. R^2 values are displayed for each relationship, indicating the strength of the fit between thermal tolerance and the variables. Dashed lines denote non-significant relationships ($p < 0.05$).

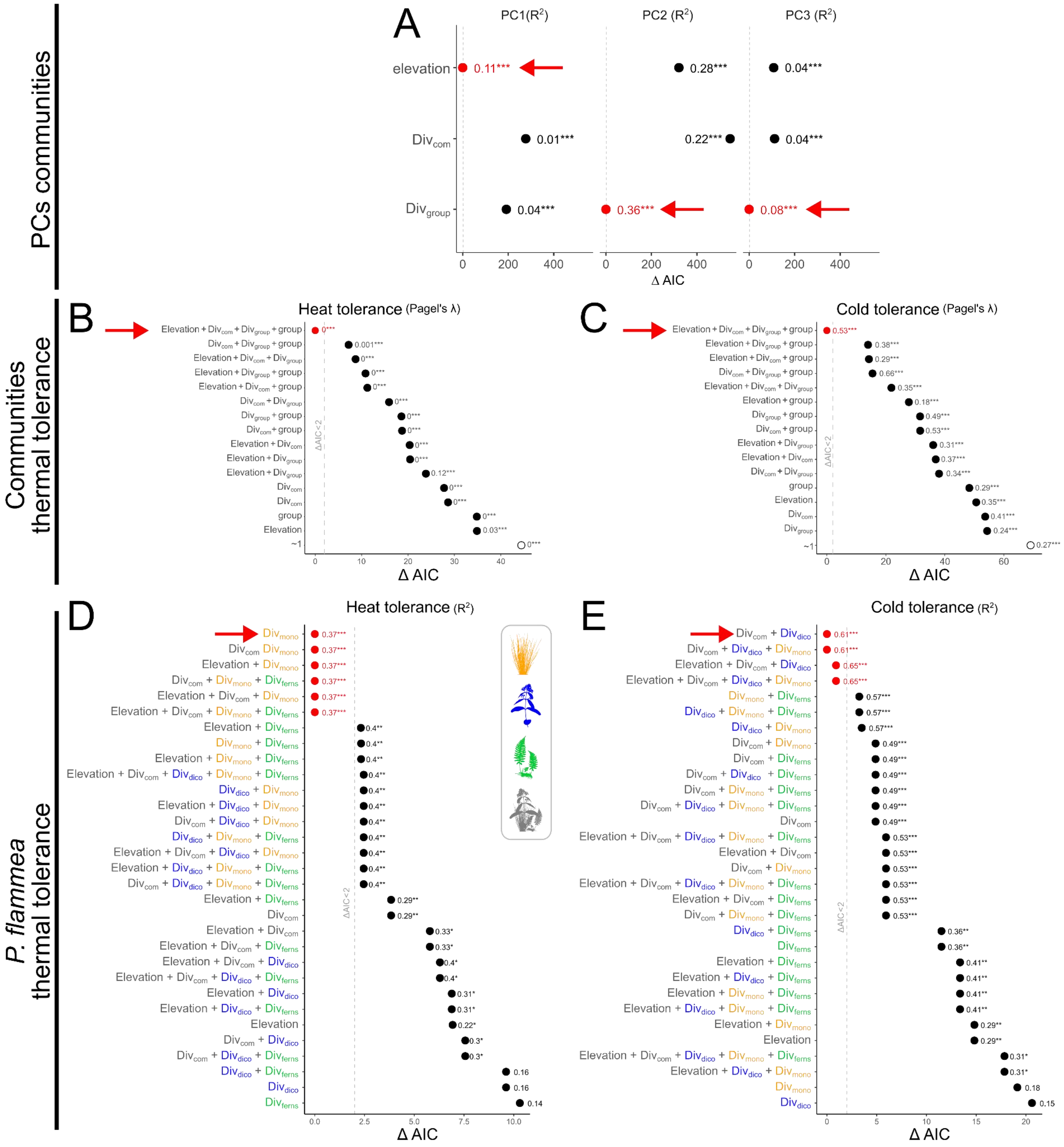

Figure S4. AIC-based model selection for trait and thermal-tolerance variation. Panels A-E show candidate models predicting (A) general trait variation (PC1–PC3); (B-C) species-level thermal tolerance (T_{50}heat and T_{50}cold) across all species except *P. flammea*; (D-E) and thermal tolerance across *P. flammea* populations. In A, we compared elevation, overall Shannon diversity (Div$_{com}$), and group-specific Shannon diversity (Div$_{group}$) as sole predictors; R^2 are indicate beside each model, red points highlight models with ΔAIC < 2, and red arrows indicate the model among them with the fewest parameters. In B-C, phylogenetic generalized linear mixed models (pGLMMs) also include plant-group identity, and Pagel's λ beside each point denotes phylogenetic signal; empty symbols are null models (to test for the phylogenetic effect alone). Panels D-E focus on *P. flammea* populations, testing all combinations of elevation and Shannon diversity, with group-specific diversity split into monocots (Div$_{mono}$, orange), dicots (Div$_{dico}$, blue) and ferns (Div$_{fern}$, green). Values, again, indicate R^2. Significance levels (* $p < 0.05$; ** $p < 0.01$; *** $p < 0.001$).$p < 0.01$), and *** ($p < 0.001$).
